# Vesicular HMGB1 release from neurons stressed with spreading depolarization enables confined inflammatory signaling to astrocytes

**DOI:** 10.1186/s12974-023-02977-6

**Published:** 2023-12-11

**Authors:** Zeynep Kaya, Nevin Belder, Melike Sever-Bahcekapili, Buket Donmez-Demir, Şefik Evren Erdener, Naz Bozbeyoglu, Canan Bagci, Emine Eren-Kocak, Muge Yemisci, Hulya Karatas, Esra Erdemli, Ihsan Gursel, Turgay Dalkara

**Affiliations:** 1 Institute of Neurological Sciences and Psychiatry, Hacettepe University, Sıhhiye, Ankara, Turkey; 2 Present Address: Biotechnology Institute, Ankara University, Ankara, Turkey; 3 Department of Molecular Biology and Genetics, Science Faculty, Bilkent University, Ankara, Turkey; 4 Present Address: Department of Biomedical Engineering, Faculty of Engineering and Natural Sciences, Bahçeşehir University, Istanbul, Turkey; 5 Department of Histology and Embryology, Faculty of Medicine, Ankara University, Ankara, Turkey; 6 Present Address: Izmir Biomedicine and Genome Center, Dokuz Eylul University, İzmir, Turkey

**Keywords:** Spreading depolarization, Neuronal extracellular vesicles, HMGB1, Inflammation

## Abstract

The role of high mobility group box 1 (HMGB1) in inflammation is well characterized in the immune system and in response to tissue injury. More recently, HMGB1 was also shown to initiate an “inflammatory signaling cascade” in the brain parenchyma after a mild and brief disturbance, such as cortical spreading depolarization (CSD), leading to headache. Despite substantial evidence implying a role for inflammatory signaling in prevalent neuropsychiatric disorders such as migraine and depression, how HMGB1 is released from healthy neurons and how inflammatory signaling is initiated in the absence of apparent cell injury are not well characterized. We triggered a single cortical spreading depolarization by optogenetic stimulation or pinprick in naïve Swiss albino or transgenic Thy1-ChR2-YFP and hGFAP-GFP adult mice. We evaluated HMGB1 release in brain tissue sections prepared from these mice by immunofluorescent labeling and immunoelectron microscopy. EzColocalization and Costes thresholding algorithms were used to assess the colocalization of small extracellular vesicles (sEVs) carrying HMGB1 with astrocyte or microglia processes. sEVs were also isolated from the brain after CSD, and neuron-derived sEVs were captured by CD171 (L1CAM). sEVs were characterized with flow cytometry, scanning electron microscopy, nanoparticle tracking analysis, and Western blotting. We found that HMGB1 is released mainly within sEVs from the soma of stressed neurons, which are taken up by surrounding astrocyte processes. This creates conditions for selective communication between neurons and astrocytes bypassing microglia, as evidenced by activation of the proinflammatory transcription factor NF-ĸB p65 in astrocytes but not in microglia. Transmission immunoelectron microscopy data illustrated that HMGB1 was incorporated into sEVs through endosomal mechanisms. In conclusion, proinflammatory mediators released within sEVs can induce cell-specific inflammatory signaling in the brain without activating transmembrane receptors on other cells and causing overt inflammation.

**Supplementary Information:**

The online version contains supplementary material available at 10.1186/s12974-023-02977-6.

## Introduction

Similar to other alarmins, high mobility group box 1 (HMGB1) assumes several intracellular functions, mostly nuclear, while it becomes an inflammatory mediator once released into the extracellular medium [1]. HMGB1’s role in inflammation has been well characterized in the immune system and in response to tissue injury, especially after discovering that it can actively be released from stressed cells without needing necrotic rupture of the plasma membrane [2]. For example, after acute injuries such as stroke or brain trauma, HMGB1 is released in such large amounts that its serum level increases to the extent that it can be detected in patient blood samples [3–6]. More recently, HMGB1 has also been shown to initiate an “inflammatory signaling cascade” in the brain parenchyma after a mild and brief perturbation, such as cortical spreading depolarization (CSD), that can be translated to pain-generating neurogenic inflammation in the meninges covering the brain [7–10]. CSD is approximately a minute-lasting profound depolarization of neurons and astrocytes that propagates across the cortex and causes migraine aura [11]. The CSD-induced chain of inflammatory events was proposed to be the mechanism underlying migraine with aura (MA) headache [7, 10]. Strongly supporting this hypothesis, recent PET imaging studies documented that patients suffering from frequent migraine with aura attacks exhibited inflammatory tracer uptake in the brain parenchyma, as well as meninges [12, 13]. Impressively, PET scans of some patients illustrated the copresence of tracer uptake in the symptomatic occipital lobe (aura/CSD side) and overlying meninges [13].

CSD-triggered parenchymal signaling appears to have distinctive characteristics compared to the immune response to tissue damage or pathogens, as it does not involve immunocompetent cell infiltration [14–16]. It also seems not identical to the HMGB1-induced inflammatory pain observed after peripheral nerve injuries because HMGB1 release from nerve terminals causes an overt inflammatory cellular reaction in the innervated skin or joint [17, 18]. In CSD-induced inflammatory signaling, neurons serve as stress sensors, and HMGB1-triggered inflammatory transcription in astrocytes functions as the reporter of this cellular strain [7, 19]. First described for CSD, this sterile, acellular inflammatory signaling may be a common response to functional (nondamaging) neuronal perturbations to report a nonhomeostatic parenchymal state and likely to promote restorative mechanisms such as brain-derived neurotrophic factor release from astrocytes [20–22]. It may exhibit unique characteristics different from the canonical HMGB1-triggered inflammatory response, as it does not involve apparent cellular injury. It may also play a role in the nonhomeostatic synaptic transmission (e.g., excess glutamatergic activity)-induced inflammatory response during psychological stress and depression [23]. Indeed, HMGB1 release from neurons has been shown in psychologically stressed rodents, and a sterile, acellular inflammatory reaction is well characterized in animal models of depression [24–26].

Despite these important developments in understanding the role of inflammatory signaling in prevalent neuropsychiatric disorders such as migraine and depression, how HMGB1 is released from neurons and how inflammatory signaling is initiated in the absence of apparent cell injury are not well characterized. Identification of these mechanisms is important, as they can provide further information on the pathophysiology of migraine and depression and reveal novel drug targets. Distinctive features different from the inflammatory response to tissue damage are likely because, for example, other pathogen-associated molecular patterns (PAMPs) and damage-associated molecular patterns (DAMPs) may not accompany neuronal HMGB1 release during functional perturbations to form complexes with HMGB1 in the extracellular medium to the extent they do under damage or pathogen-associated conditions [1, 27]. Therefore, we investigated how HMGB1 is released from neurons and initiates inflammatory signaling in astrocytes after CSD. We have found that HMGB1 is mainly released in extracellular vesicles, which are taken up by astrocyte processes, creating conditions for compartmentalized (selective) communication between neurons and astrocytes.

## Material and methods

### Animals and induction of CSD

Animal housing, care, and application of experimental procedures were all performed in accordance with institutional regulations as approved by the Hacettepe University Animal Experiments Local Ethics Committee (Approval numbers: 2017/10-5 and 2018/30-1). The experiments were carried out according to the Guide for the Care and Use of Laboratory Animals and reported in accordance with the ARRIVE guidelines. The animals were housed under a 12-h light–12-h dark cycle at a temperature of 22 ± 3 °C and 40–60% humidity and allowed free access to food and water.

Male Swiss albino adult mice (25–35 g) (*n* = 31) (from Hacettepe University Experimental Animal Facility), both sexes of Thy1-ChR2-YFP adult mice (*n* = 8) expressing the light-activated ion channel, channelrhodopsin-2, fused to yellow fluorescent protein under the control of the mouse thymus cell antigen 1 (Thy1) promoter [28] (stock #007612, Jackson Laboratories) and hGFAP-GFP adult mice (*n* = 7) expressing green fluorescent protein under the control of the astrocyte-specific glial fibrillary acidic protein promoter [29] (stock #003257, Jackson Laboratories) were used. Mice were anesthetized with xylazine (10 mg/kg, intraperitoneal (i.p.)) and urethane (1.25 g/kg, i.p., U2500, Sigma) or isoflurane (1.5–2%) under continuous oxygen delivery (2 l/min) and were placed in a stereotaxic frame (Digital Lab Standard Stereotaxic Frame, Stoelting). Body temperature was monitored with a rectal probe and maintained at 37.0 ± 0.2 °C by a homeothermic blanket control unit (Kent Scientific). Pulse rate and oxygen saturation were monitored by an oximeter using a mini Y-clip hind paw probe (The LifeSense^®^ VET pulse oximeter, Nonin Medical Inc.).

In Swiss albino and hGFAP-GFP mice, the parietal bone was thinned using a microdrill (Fine Science Tools, USA), and a 1.5-mm burr hole was opened over the frontal region of the right hemisphere (1 mm anterior and 1 mm lateral to bregma). The skull was irrigated with cold saline to prevent complications due to heating caused by the drilling procedure. The dura under the burr hole was kept intact and maintained moist by repeated applications of aCSF preheated to 37 °C until the experiment started. A Ag–AgCl pellet electrode was placed over the thinned parietal bone to record the direct current (DC) potential changes. EEG gel was applied to the electrode tip to enhance electrical contact with bone. A reference electrode was placed between the layers of the neck muscles. DC potential changes, heart rate, and tissue oxygen saturation were recorded using the Lab Chart data acquisition system (AD Instruments). A single CSD was induced by pinpricking the frontal cortex and verified with the DC potential shift observed.

For optogenetic stimulation experiments in Thy1-ChR2-YFP mice, the CSD waves were triggered by optogenetic stimulation (450 nm) delivered by a fiberoptic probe, which was positioned and secured over the skull (the same location where the cortex is pinpricked) by a cable holder as previously described by Houben et al. [30]. No burr hole drilling or thinning was performed in the skull for optogenetic stimulation to ensure minimal invasiveness. The optical fiber, 400 μm in diameter, with a numerical aperture of 0.48, was in full contact with the skull. A suprathreshold light stimulus of 50 mJ was continuously applied for 10 s to trigger each CSD. The laser light was turned off upon completion of the 10-s stimulus. This stimulation protocol has previously been optimized for our laboratory and reliably results in a CSD for every application.

### Immunofluorescent labeling

Mice were deeply anesthetized and transcardially perfused with 0.04% heparinized saline and 4% paraformaldehyde (PFA). The brains were quickly removed, postfixed in the same PFA solution overnight, and cryoprotected in 30% sucrose solution for two days. Eight-micron-thick coronal sections were cut on a freezing cryostat (CM1100, Leica GmbH). Sections were blocked with either 10% normal goat serum or 3% bovine serum albumin (BSA) in phosphate-buffered saline (PBS) and immunostained with antibodies against HMGB1 (1:200, ab18256, Abcam), CD171 (1:200, ab24345, Abcam), Iba1 (1:200, 019-19741, Fuji Film), GFP (1:200, ab1218, Abcam), S100β (1:200, ab52642, Abcam), and NF-ĸB p65 (1:200, 8242, Cell Signaling Technology) at + 4 °C, followed by secondary labeling with goat anti-rabbit Cy2 or Cy3 at room temperature (RT) (1:200, 115-225-146 or 115-165-146, respectively, Jackson Immunoresearch). Double labeling (CD171-HMGB1, Iba1-p65, and S100β-p65) was carried out with incubation of primary antibodies either simultaneously or consecutively. The sections were mounted in glycerol/PBS (1:1) medium containing 12.5 mg/ml sodium azide and 1 µl/ml Hoechst-33258 (H3569, Thermo Fisher Scientific) for nuclear identification. Primary antibody omission incubations with blocking solution were performed to test the specificity of immunoreactivity. All sections were examined under a laser scanning confocal microscope (SP8, Leica GmbH) with appropriate filter sets.

### Isolation of extracellular vesicles

Mice were deeply anesthetized, and to reduce any other extracellular vesicle (EV) release due to tissue processing, the cortices were dissected using sterile, ice-cold tools after quickly harvesting brains on ice. The harvesting procedure was completed in less than 2 min. The cortices were dissociated with a gentleMACS dissociator (130-093-235, Miltenyi Biotec) in cold PBS in 30–40 s. To obtain only extracellular fluid, intact cells were removed by centrifuging at + 4 °C for 10 min at 1500 *g*. The supernatants from cortex samples were once again centrifuged at + 4 °C for 30 min at 10,000 × g to remove large vesicles and cell debris. The final supernatants obtained were processed with the Total Exosome Isolation Kit (from plasma) (4484450, Thermo Fisher Scientific) according to minimally modified instructions. EVs were resuspended in PBS and rotated at 360° overnight at + 4 °C to separate small EVs from polyethylene glycol. Finally, the EV suspensions were flash frozen in liquid nitrogen and kept at − 80 °C until further processing.

### Characterization of EVs using flow cytometry

Isolated EVs were characterized by a bead-based detection method as previously reported with some modifications [31]. Briefly, 10 μl carboxyl-modified latex beads (bead diameter: 3.9 μm, C37278, Thermo Fisher Scientific) were incubated with 10 μg purified anti-mouse CD63 or CD81 antibodies (clones NVG-2, EAT-2, respectively, Biolegend) overnight on a rotator at a low speed at RT. Subsequently, the bead/antibody complex was precipitated by centrifugation at 2000 × g and blocked with 5% BSA containing PBS for 6 h at RT. The coated and blocked beads were washed with PBS by centrifuging at 2000 × g for 10 min at RT and resuspended in 1% BSA containing PBS. For each labeling of EVs, 1 μg protein containing intact EVs was mixed with 1 μL of the final anti-CD63- or CD81-coated bead solution in PBS with gentle mixing for 30 min at RT. The EV-bound beads were centrifuged at 2000 × g for 10 min, and the supernatant was removed. Next, the EV/bead complex was incubated with anti-CD63-PE or anti-CD81-PE antibodies (134904, 104906, respectively, Biolegend) in PBS for 30 min with gentle mixing at RT. At the end of the incubation period with fluorescent-tagged antibodies, the EV/bead complexes were centrifuged, washed twice, and analyzed by a NovoCyte flow cytometer (Acea Biosciences). Isotype antibodies were used to evaluate the specificity of each label.

The particles were counted after a proper FSC-SSC gating strategy to exclude debris and clumps (Additional file 1: Fig. S1). The percentage of antibody-positive beads and their mean fluorescent intensity were used for comparison. Each EV sample was stained in triplicate, and the mean percentage of each sample was used in the analysis.

### Biophysical characterization of EVs with scanning electron microscopy

The EV sample preparation was diluted 1:10,000 in ddH_2_O, and then 10 μL was left to dry at RT on a slide attached to a stub with carbon tape before being coated with 4-nm gold palladium. Scanning electron microscopy (SEM) images were acquired using a GAIA3 FIB-SEM (Tescan, Czech Republic) operated at 4–5 kV and a scan speed of 6 to 7.

### Nanoparticle tracking analysis

Nanoparticle tracking analysis (NTA) was performed on EV samples that were diluted to 1 μg/µL in PBS to estimate size distribution and particle concentration. A tunable pulse resistive index system (qNano, Izon Biosciences) was used for the analysis.

### Neuron-derived EV enrichment

Neuron-derived EVs were captured by way of CD171 (L1CAM) expressed on their surface [32–35]. Latex beads were coated with purified anti-CD171 antibody (130-115-812, Miltenyi Biotec) as described above. One microliter of coated latex beads was incubated with 3 µg protein containing intact mixed exosomes extracted from brain samples. After incubation, the exosome–bead complex was precipitated at 10,000 × g for 5 min, and the pellets were resuspended in PBS.

### Western blotting

Pelleted EV–bead complexes were resuspended in radioimmunoprecipitation assay buffer. The protein concentration of all lysates was determined by a BCA assay (23227, Thermo Fisher Scientific). Samples and albumin standards were prepared as instructed by the manufacturer, and their absorbance at 562 nm was measured with a spectrophotometer (Infinite F50, Tecan). Equal amounts of protein were loaded on 12% SDS‒PAGE gels and subsequently transferred to PVDF membranes by semidry transfer. Nonspecific protein binding was blocked by incubating the PVDF membranes in 5% skim milk powder for an hour, and then the membranes were incubated with antibodies against HMGB1 (1:500, ab18256, Abcam), TSG101 (1:500, ab125011, Abcam), CD171 (1:500, ab24345, Abcam), ALDH1L1 (1:500, ab87117, Abcam), S100β (1:1000, ab52642, Abcam), and Iba-1 (1:1000, NB100-1028, Novus Biologicals) overnight at + 4 °C. The next day, after washing, the membrane was incubated with horseradish peroxidase-conjugated goat anti-mouse or anti-rabbit IgG secondary antibody (1:5000, ab6789 or ab6721, respectively, Abcam) for 2.5 h at RT. The protein bands were visualized by the chemiluminescence method (34094, Super Signal West-Femto, Thermo Fisher), and the images were captured by Image Station 4000 (Kodak). FIJI/ImageJ (National Institute of Health, USA) was used for densitometric analysis. Signals were normalized to TSG101 intensities as a marker of the total EV amount. Raw blot images are demonstrated in Additional file 2: Fig. S2.

### Immunoelectron microscopy

After performing cardiac perfusion with 2.5% glutaraldehyde solution, the cortices were sliced into 1 × 5-mm-thick sections. Slices were fixed overnight with 2.5% glutaraldehyde and 2% PFA in Sorenson phosphate buffer (SPB, 0.2 M, pH: 7.2). Slices washed with SBP (0.1 M) were then fixed for a second time with osmium tetroxide (1%, O5500, Merck) for 2 h. The slices were then washed with SBP. After staining with uranyl acetate and the third fixation, dehydration was performed by passing through graduated ethanol. Following these steps, blocks were obtained by polymerization in araldite CY 2012 embedding material. Then, 800 nm semi-thin sections were taken from the blocks by ultramicrotome (Ultracut R, Leica GmbH) into nickel grids after appropriate regions were determined. Thin sections were incubated with saturated sodium metaperiodate (106597, Merck) at RT for 30 min to melt the araldite. The grid was subsequently incubated with normal goat serum for 10 min to prevent background signal at RT. The sections were then incubated with anti-HMGB1 antibody (1:100, ab18256, Abcam) overnight. After washing with Tris buffer, sections were incubated with 5- or 10-nm colloidal gold-conjugated goat anti-rabbit antibody (1:20, A-31565 or A-31566, respectively, Thermo Fisher Scientific) for an hour, followed by washing again with Tris buffer. The grids carrying the sections were stained with uranyl acetate and lead citrate at the final stage. The sections were examined by a transmission electron microscope (Leo 906 E (80 kV), Oberkochen).

### Assessing HMGB1-loaded EV uptake by astrocytes and microglia

All image-processing steps were performed using FIJI/ImageJ (v2.9.0/1.53t, National Institute of Health). Fifteen-µm-thick image stacks of ~ 50 × 50 µm and 1024 × 1024 pixels and 0.3 µm Z-steps were background-subtracted with a rolling ball radius of 70 pixels (for the green-Cy2-GFP/red-Cy3-Iba1 channel) or 20 pixels (for the red-Cy3/green-Cy2-HMGB1 channel) and then despeckled and denoised with a theta of 20 pixels using stock functions of FIJI/ImageJ. The entire stack was then subdivided into three 5-µm-thick substacks, and maximum intensity projections (MIPs) were generated for further analysis. Cy2-GFP-positive astrocyte processes were visually identified for subsequent analysis. For each MIP image, three to five processes were selected that did not overlap with the adjacent MIPs. That made six to ten processes for each examined astrocyte, taking all MIPs into account. Astrocytes and microglia with fewer than 6 identifiable processes were excluded from the analysis. For each process to be analyzed, a 5-µm-thick region was manually traced using the freehand ROI selection tool. At least 50% of the selected pixels were outside the analyzed process. For each process ROI, the EzColocalization algorithm of FIJI [36] was used to calculate a threshold overlap score (TOS) with the Costes thresholding algorithm [37], allowing an unbiased, systematic and quantitative evaluation of whether the two markers (GFP/Iba1 and HMGB1) were colocalized in comparison to uniformly distributed random signals. TOS scores are distributed between − 1 and 1, where 1 indicates absolute maximum colocalization, − 1 indicates absolute maximum anticolocalization, and 0 indicates random overlap between two signals with no colocalization. For three-dimensional volume reconstruction in Fig. 3I, first the 15-µm Z-stack of an astrocyte (with green and red imaging channels) was resampled axially for isotropic voxel spacing. Then, the image was binarized independently for the red and green channels, using the default thresholding algorithm of FIJI/ImageJ. Binary Z stacks were imported to Vaa3D environment [38] and surface mesh was generated from the volume data, using the marching cubes method and a mesh type of label field surface with a mesh density of 100. These surface data for both green and red channels were saved in.obj format and imported into Paraview 5.11.1 for visualization. During 3D visualization, the opacity of the green channel surface was set at 50% to make the HMGB1 puncta visible within the GFP-positive process.

### Statistical analysis

All statistical analyses were performed using SPSS Statistics v25 (IBM). After determining that the data were not distributed normally, the Mann‒Whitney U test was used to compare two groups.

## Results

**Fig. 1:**
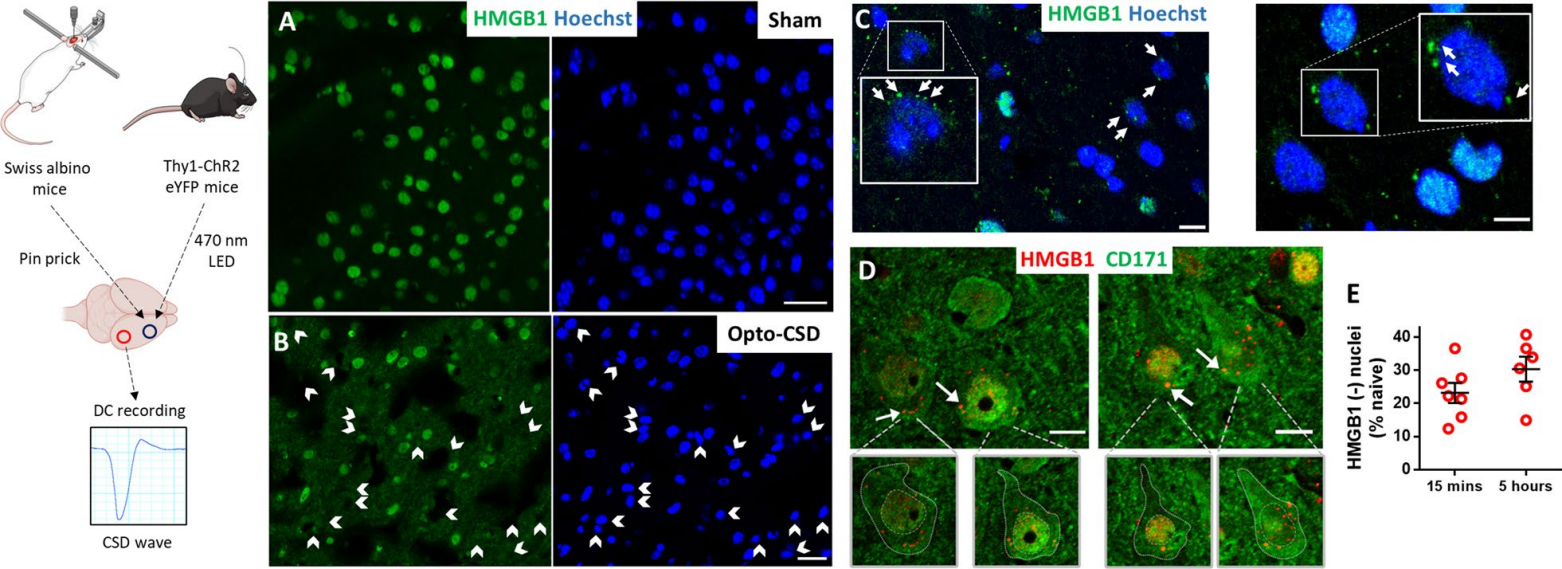
CSD causes the release of HMGB1-immunopositive puncta from neuronal nuclei. CSD was triggered by pinpricking the frontal cortex in wild-type mice under anesthesia or by depolarization of cortical neurons with a blue LED light through the intact skull in freely moving Thy1-ChR2 eYFP mice. The ignition of each CSD wave was confirmed by recording the negative DC current shift at a location posterior to the stimulation site (left). **A** Most nuclei (labeled blue with Hoechst) were immunopositive for HMGB1 (green) in the cortex of sham-operated mice (**A**), while CSD caused the loss of nuclear HMGB1 labeling (white arrowheads) in neurons within 15 min (**B–D**). **B** CSD triggered by noninvasive optogenetic stimulation induced HMGB1 release similarly to pinprick-triggered CSD in wild-type mice (**C, D**). Cells that lost nuclear HMGB1 immunopositivity are marked with arrowheads in the green channel in parallel with their corresponding nuclei labeled with Hoechst in blue channel. **C** HMGB1-positive puncta (green) scattered out of the nucleus (blue) were visible in neurons that lost most of their nuclear HMGB1 labeling (white arrows). **D** Many HMGB1-positive puncta (red, white arrows) were present within the cytoplasm around the nuclei of cortical neurons identified by CD171 immunolabeling (green). Boundaries of neuronal cytoplasm and nucleus are delineated in the insets below to better illustrate distribution of the puncta. Shedding of HMGB1-labeled puncta from cells that lost their nuclear HMGB1 immunopositivity (completely or partially) was noticeable as soon as 15 min after CSD. White arrows in C and D mark puncta in proximity to the nuclei, suggesting that HMGB1 is released in vesicles. **E** The ratio of HMGB1-immunonegative nuclei (% of naïve Hoechst-positive nuclei) did not increase from 15 min to 5 h after CSD (*p* = 0.23, Mann–Whitney U test), suggesting that HMGB1 is released at once right after CSD. Insets are magnified images of the boxed areas. Images are maximum projections of confocal z-stacks. Scale bars: 10 µm

To elucidate how HMGB1 is released and activates inflammatory signaling in astrocytes, we subjected wild-type mice (*n* = 31) or Thy1-ChR2-YFP transgenic mice (*n* = 8) to a single CSD evoked by pinprick or optogenetic stimulation. While most of the nuclei contained HMGB1 in sham-operated mice (Fig. 1A), optogenetically triggered CSD induced HMGB1 release (Fig. 1B), similar to that seen in wild-type mice subjected to pin-prick-triggered CSD (Fig. 1C, D), confirming that HMGB1 release was caused by CSD but not pinprick-induced trauma at the application site 2–3 mm away. Hence, we performed most of the experiments on wild-type Swiss albino mice. We induced a single CSD but not multiple CSDs because most migraine headaches are preceded by a single short-lasting aura [39, 40]. Furthermore, experimentally, multiple CSDs activate microglia unlike a single CSD and potentially may involve signaling pathways not seen after typical sporadic aura but might be more reminiscent of frequent MA attacks [8, 41–44].

In line with previously published studies from our laboratory showing that HMGB1 was released mainly from NeuN-positive neurons [7, 10], we confirmed that most of the nuclei that lost HMBG1 immunostaining 15 min after CSD were neurons identified this time with CD171 (L1CAM) immunolabeling (Fig. 1D). Interestingly, we noticed many HMGB1-positive small puncta within and around neurons that lost their nuclear HMGB1 immunostaining (Fig. 1C, D). These puncta were located on the nucleus–cytoplasm border and in the neuropil soon after CSD (i.e., at the earliest time that the brain could be perfusion fixed after CSD, which is approximately 10–15 min). The ratio of HMGB1 immunonegative nuclei remained unchanged from 15 min (23 ± 3.0% of naïve) to 5 h (30 ± 3.8% of naïve, mean ± SE) after CSD (*p* = 0.23, Mann–Whitney U test) (Fig. 1E).

**Fig. 2:**
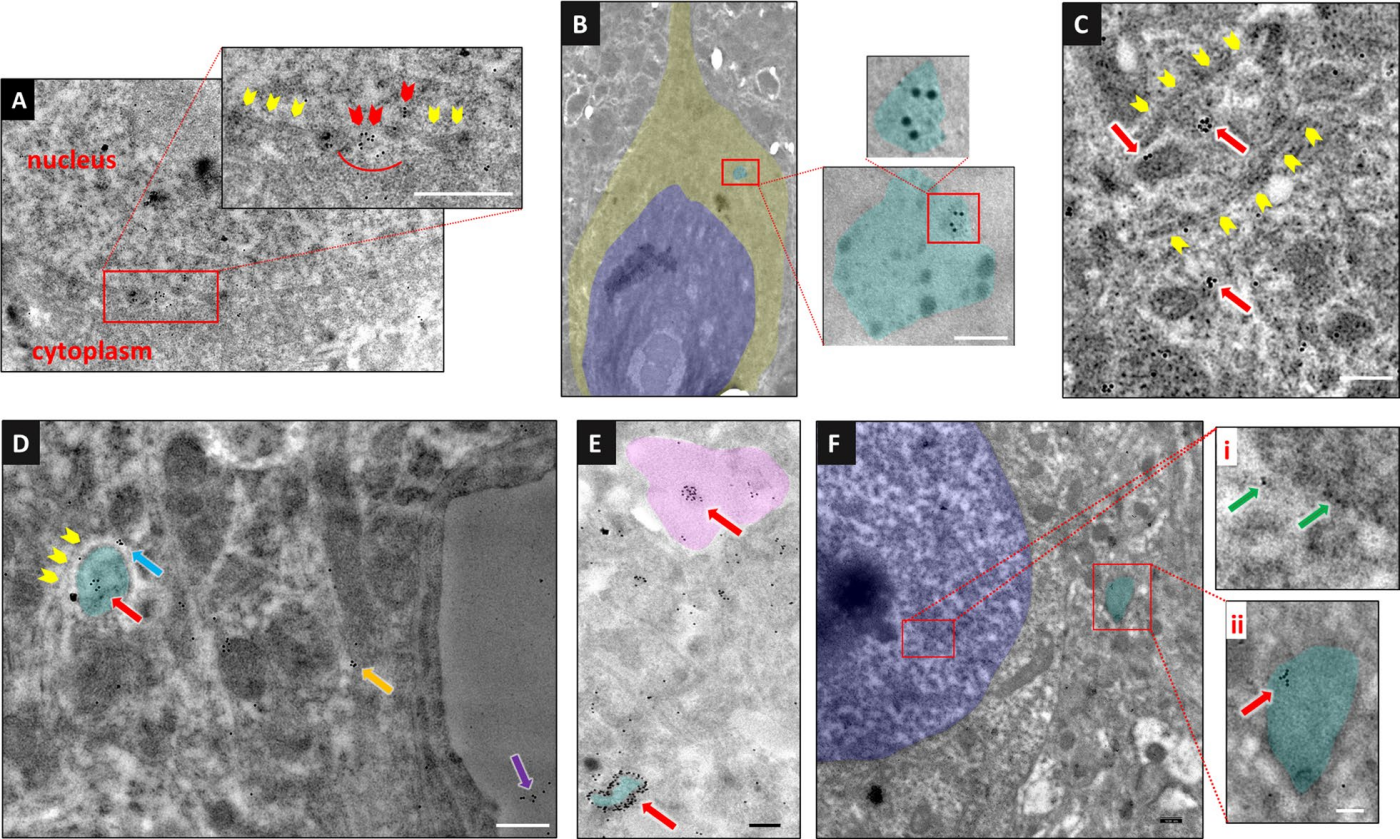
Immunoelectron microscopy illustrates that HMGB1 is released within small EVs. HMGB1 was labeled using 10 nm diameter gold-conjugated secondary antibodies on a perfusion-fixed brain section 15 min (**A–E**) and 3 h (**F**) after CSD. **A** The magnified image of a neuronal nucleus and cytoplasm in the inset shows gathering of gold particles (HMGB1) in areas where the nuclear membrane is not visualized (red arrowheads), suggesting that HMGB1 passes to the cytoplasm through nuclear pores, whereas no such accumulation is observed in the regions where the nuclear membrane is clearly visible (yellow arrowheads). **B** In another neuron, a multivesicular body (light blue) containing several sEVs, one of which harbors gold particles marking HMGB1, is seen. In this image, the focus was set on the gold particles to emphasize them at the expense of partly losing the details of cellular structures including the MVB membrane. However, the MVB can easily be identified by its more electrodense cytoplasm. **C** sEVs bearing HMGB1 molecules (red arrows) are seen within an astrocyte process (upper left) and the neighboring neuron soma (lower right). The plasma membranes of the neuron and astrocyte processes abutting each other (yellow arrowheads) are clearly visible. **D** The clustering of HMGB1 molecules (red arrow) presumably in an sEV inside an astrocyte process (light blue) abutting a neuron soma and in another sEV (blue arrow) between the neuron and astrocyte process are noticed. The plasma membrane of the neuron (yellow arrowheads) and the astrocyte process are visible. On the right, clustered HMGB1 molecules (possibly packed in a sEV) are also seen in an astrocyte end foot (orange arrow) and capillary lumen (purple arrow). **E** A sEV bearing clustered HMGB1 molecules (red arrow) within the tip of an astrocyte process (pink) and large numbers of gold particles lining the outline of an endosome (light blue) are easily visible. In this image, the focus was set on the gold particles as in panel B. **F** sEVs containing HMGB1 were still present in the cortex 3 h after CSD. Whereas gold-conjugated HMGB1-positive particles are scattered inside the nucleus of a neuron (i, green arrows), clustered gold particles (red arrow) are seen in a cytoplasmic vesicle (endosome, ii, light blue). The scale bars are 100 nm

In light of the studies showing that HMGB1 is released from intact, nonnecrotic cells by the vesicular pathway in myeloid cells and hepatocytes [45–48] rather than the endoplasmic reticulum–Golgi-dependent pathway due to the lack of a leader peptide sequence [49, 50], we hypothesized that the HMGB1-immunoreactive puncta might be groups of EVs, transporting HMGB1 from the cytoplasm to the extracellular medium. Fluorescence emitted by immunolabeled EV clusters as well as by EVs bound to multiple fluorescent-tagged antibodies is within the optical resolution of confocal microscopy, although single EVs are too small to be detected by light microscopy [51]. To investigate this possibility, we immunolabeled HMGB1 in brain sections obtained 15 min and 3 h after CSD using a colloidal gold-conjugated secondary antibody and examined the sections with transmission electron microscopy (Fig. 2). The gold particles attached to HMGB1 were concentrated under the nuclear membrane adjacent to the nuclear pores in sections prepared 15 min after CSD (Fig. 2A). Several of them were spotted within the pores themselves. In addition, several gold particles lining the outline of the endosomes and within the multivesicular bodies were detected in the cytoplasm (Fig. 2B, and E). At 15 min and 3 h, gold particles were also seen on the wall and interior of vesicles. Gold particles formed clusters generally smaller than < 100 nm (in the range of exosomes) within the cytoplasm of neurons and adjacent astrocyte processes (Fig. 2C–F). Although we observed scattered gold particles attached to the chromatin (Fig. 2Fi), residual nuclear HMGB1 was not commonly detectable with immunofluorescence, perhaps due to the low intensity of the signal. However, partial immunopositivity located at the periphery of the nucleus was occasionally observed at the 15-min time point (Fig. 1C, D). It is unclear whether CSD could cause the vast majority of HMGB1 to leave the neuronal nucleus, as the immunostaining findings suggest. One possibility is that the antibody may not be recognizing the altered structure of residual HMGB1 in the nucleus (e.g., due to formation of disulfide bonds [52, 53]), which remains to be clarified with non-antibody-based methods (e.g., mass spectrometry).

**Fig. 3:**
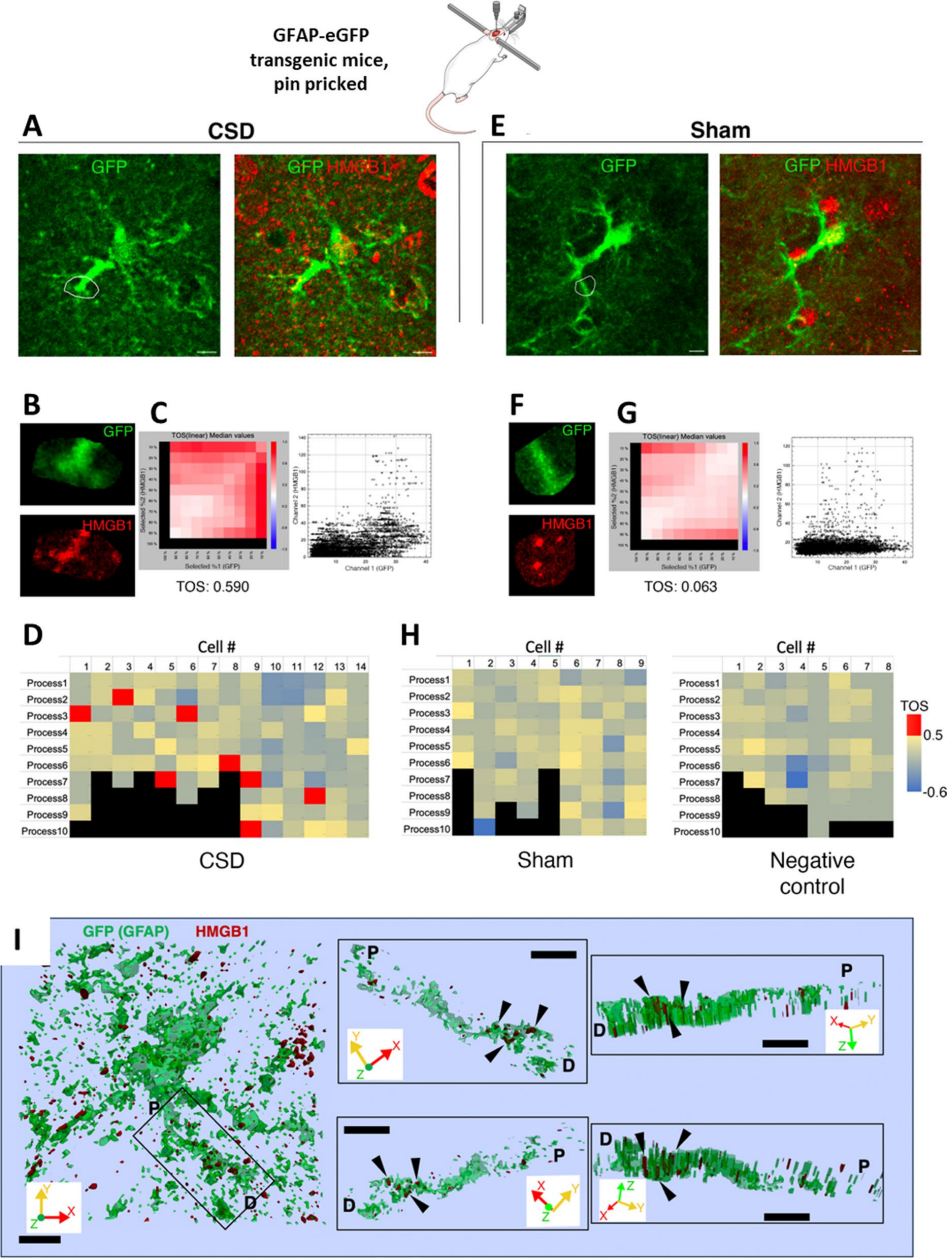
EVs loaded with HMGB1 are taken up by astrocyte processes. To assess the prevalence of HMGB1-loaded EV uptake by astrocytes, we examined the colocalization of HMGB1-immunopositive puncta with astrocyte processes visualized with anti-GFP antibodies in transgenic mice expressing GFAP-GFP and subjected to CSD. **A** and **E** show two representative cells from the CSD and Sham groups, with zoomed-in views of the outlined regions of interest (ROIs) (**B** and **F**). Scale bars: 10 µm. Scatter plots (**C**, **G**) show the distribution of corresponding pixel intensities in GFP and HMGB1 channels in the indicated ROIs. Metric matrices for threshold overlap scores (TOS) are shown in **D**, **H** with top percentages of highest intensity pixels in each ROI indicated in columns and rows for GFP and HMGB1, respectively. Each cell with color code in the matrix shows TOS values when the corresponding percentage of highest intensity pixels are selected in each channel, + 1.0 indicating absolute correlation, -1.0 indicating absolute anti-correlation, and 0 indicating random distribution and overlap between two channels. TOS values are not informative when one threshold is 100%; hence, the left column and bottom row are shown in black. Despite heterogeneity in the HMGB1 content of astrocyte processes and the presence of nonspecific punctate signals, the EzColocalization algorithm of FIJI disclosed that in the CSD cortex (*n* = 3 animals), 7 out of 14 astrocytes had at least one process with a TOS above 0.5, indicating a strong positive colocalization of HMGB1 and GFP signals, while none of the 17 astrocytes analyzed from sham (*n* = 2) and negative staining control animals (*n* = 2) was over that threshold, suggesting that uptake of released HMGB1-loaded EVs by astrocytes was not uncommon. **I** 3D surface reconstruction of a GFP-positive astrocyte and its process shows that HMGB1 immunopositive puncta (black triangles) are located inside the process but not superimposed falsely due to intensity projections. Black rectangle on the left panel indicates the HMGB1-immunopositive process that is visualized on the right panels from different angles in 3D. P and D indicate the proximal and distal ends of the process, respectively. Scale bars: 2 µm. X, Y, and Z axes of the volume are shown for orientation

**Fig. 4:**
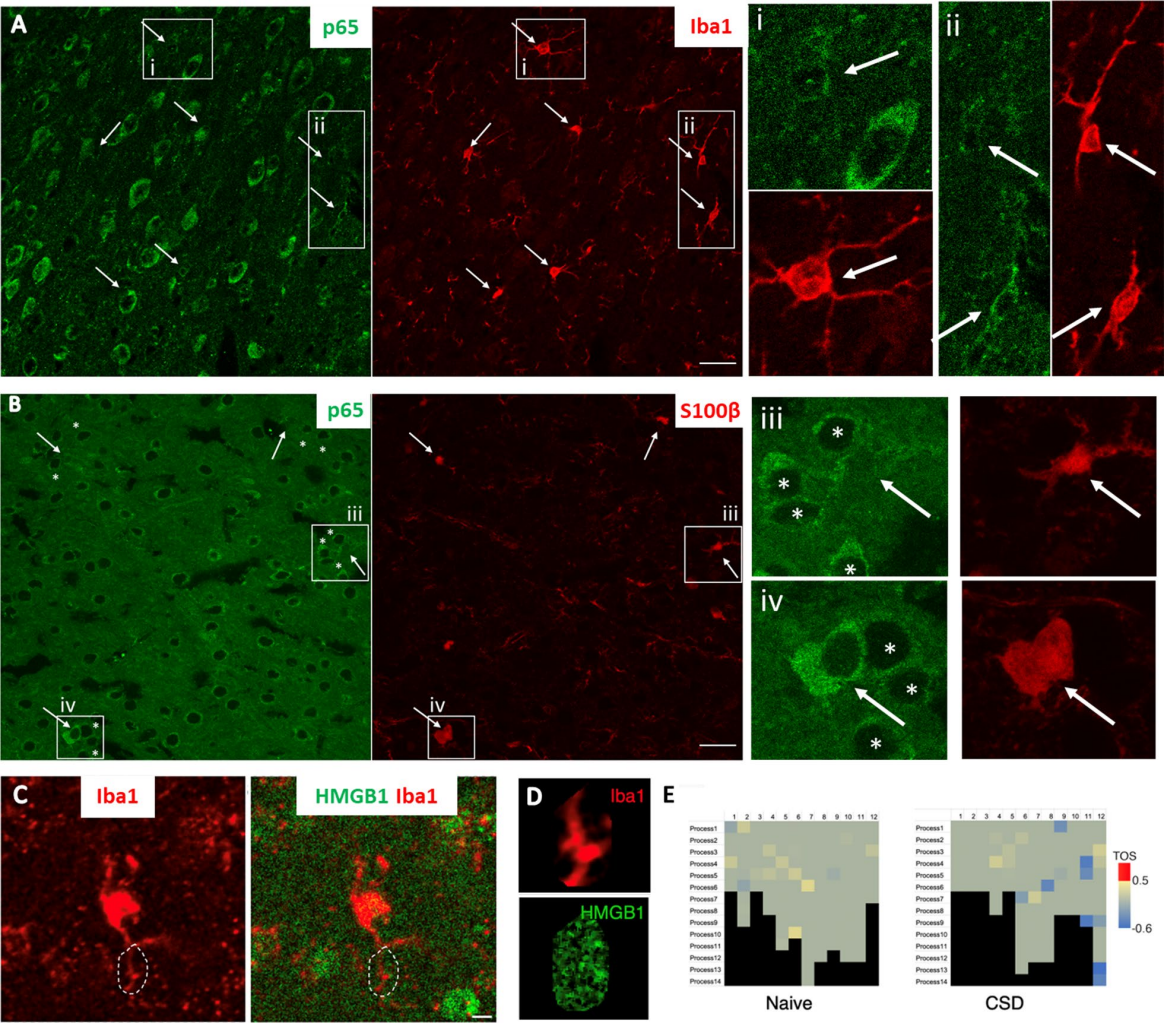
EVs loaded with HMGB1 are not observed in microglial processes. **A** No nuclear translocation of NF-ĸB p65 (green), a marker of proinflammatory transcriptional activity, was detected in microglia (Iba-1 immunopositive, red, arrows) 24 h after CSD. The round cell body and thin, long processes of microglia (arrow) also indicate that they are in an inactive state. Magnified images on the right of the boxed areas (i and ii, corresponding images in green and red) illustrate the presence of perinuclear (cytoplasmic) but not nuclear NF-ĸB p65 (green) in microglia (red, arrows). **B** Unlike microglia, S100β-positive astrocytes (red, arrows) exhibited NF-ĸB p65 nuclear positivity (green) shortly after CSD. iii and iv (corresponding images in green and red) depict p65-positive nuclei (light green) of S100β-labeled astrocytes (arrows), in contrast to non-astrocytic neighbors with p65 immunonegative nuclei (asterisks). **C** To assess whether HMGB1 was taken up by microglial processes 1 h after CSD, we examined the colocalization of HMGB1 immunopositive puncta with microglial processes visualized by anti-Iba1 antibodies. **C** Shows representative microglia 1 h after CSD, with zoomed-in views of the outlined ROI in **D**, scale bar: 5 µm. **E** Analysis using the FIJI EzColocalization algorithm revealed that in the cortex subjected to CSD (*n* = 3) and in naïve animals (*n* = 3), none of the 24 microglia analyzed had any processes with a threshold overlap score greater than 0.5, indicating a lack of colocalization between the HMGB1 signal and microglial processes

To assess the prevalence of EV uptake by astrocytes, we examined the colocalization of HMGB1-immunopositive puncta with astrocyte processes visualized with anti-GFP antibodies in brain sections from hGFAP-GFP-expressing mice subjected to a single CSD (Fig. 3). When analyzed with an algorithm that can identify positive colocalizations scoring higher than random chance, we found significant colocalization, suggesting that the uptake of released HMGB1-carrying EVs by astrocytes was not uncommon. Despite heterogeneity of the distribution of astrocyte processes with HMGB1-immunopositive puncta in a selected imaging plane from the cortex subjected to CSD (*n* = 3 mice), 7 out of 14 astrocytes analyzed had at least one process with a median threshold overlap score (TOS) above 0.5, indicating a strong positive colocalization of the HMGB1 signal and astrocyte marker, while none of the 16 astrocytes analyzed from sham and negative staining control animals (*n* = 4 mice) exceeded this threshold. Previous studies from our laboratory detected that HMGB1 release triggered NF-ĸB p65 nuclear translocation in 82–88% of cortical astrocytes [7, 10]. Conforming to these findings, we commonly observed NF-ĸB p65 nuclear translocation in astrocytes but not in microglia (Fig. 4A and B). The number of NF-ĸB p65-positive nuclei in Iba1-positive cortical microglia was 1 out of 121 (total count) in naïve mice (*n* = 3) and 4 out of 121 (total count) one hour after CSD (*n* = 3 mice). In accordance with this, none of the Iba1-positive processes of 12 microglia analyzed 1 h after a single CSD exhibited significant colocalization with HMGB1-immunopositive puncta as for the 12 microglia analyzed from control mice (*n* = 3 per group, Fig. 4C–E). Consistent with previous studies revealing that microglia are activated only 24 h after multiple CSDs [8, 42–44, 54], we did not observe morphological changes in microglia 24 h after a single CSD either (Fig. 4A). Taken together, these data suggest that the HMGB1-carrying small EVs (sEVs) released from neurons are preferentially taken up by astrocyte processes that extensively cover neuronal soma [55–57].

**Fig. 5:**
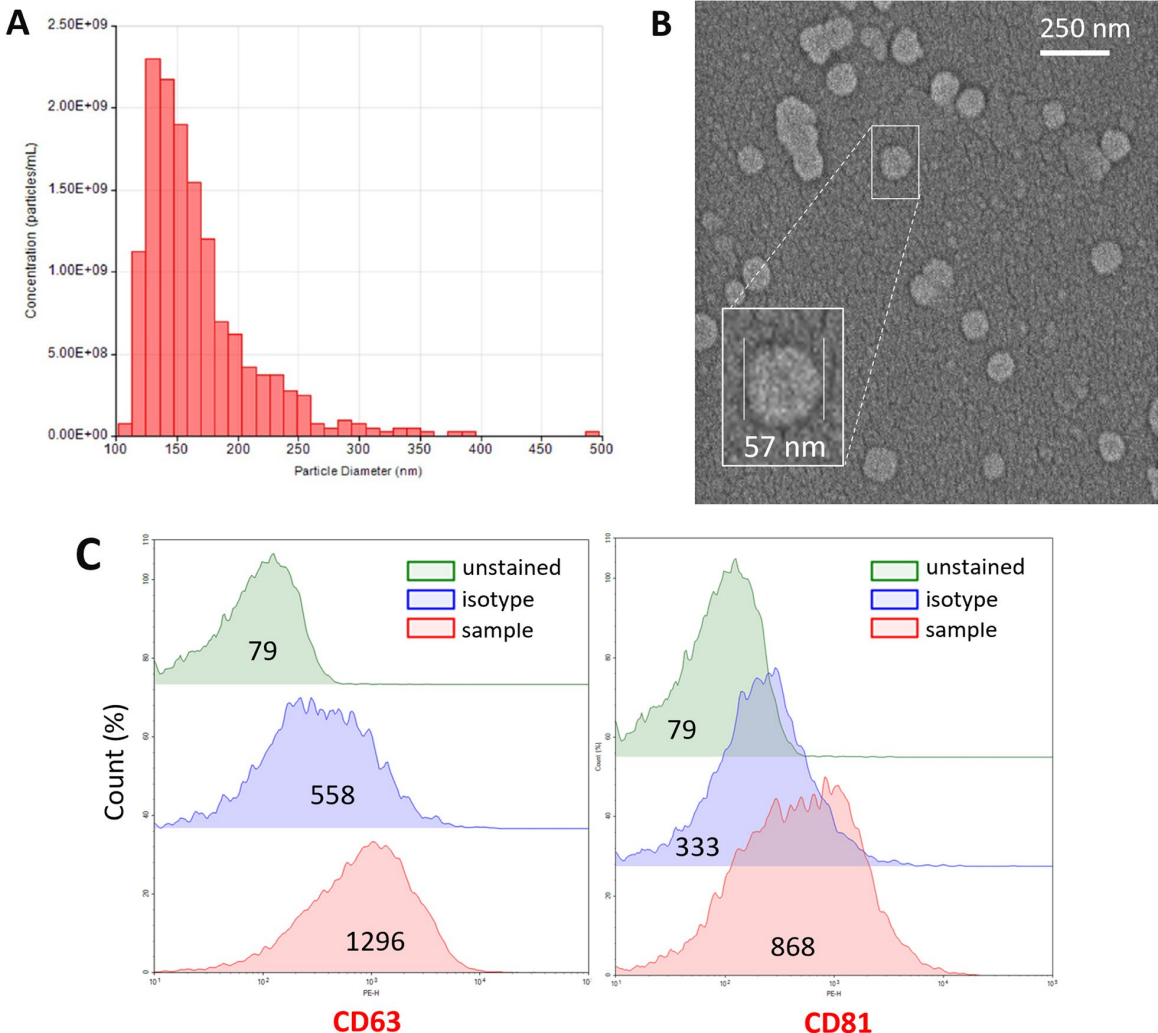
Characteristics of the extracellular vesicles released by CSD. EVs isolated from the brain one hour after CSD were evaluated in accordance with MISEV guidelines. **A** Nanoparticle tracking assay showed that the mean particle diameter was 167 nm, compatible with sEVs (SD = 46.6 nm; d90/d10 = 1.82). The concentration was 1.39e + 10 particles/ml. **B** SEM image of EVs derived from the mouse brain cortex and a magnified image of a single EV with a 57 nm radius from the same sample (inset). **C** Flow cytometric analysis of EVs captured with anti-CD63 antibody-coated latex beads and labeled with anti-CD63 and anti-CD81 antibodies verified that they expressed typical EV surface markers. Each sample was compared with an unstained sample and with a sample labeled with an isotype antibody. The numbers in the middle of each histogram show the mean fluorescence intensity

**Fig. 6:**
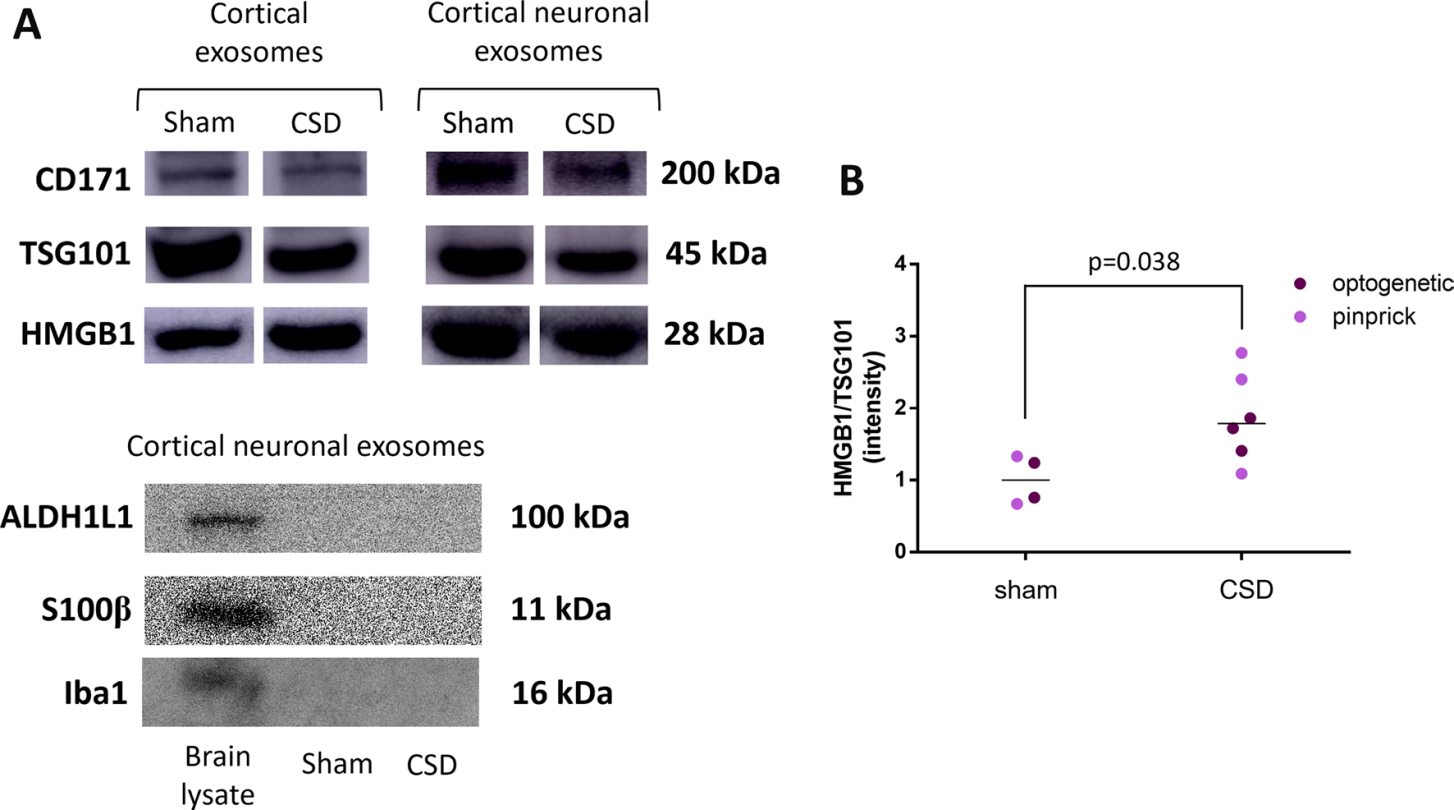
EVs isolated from the cortex after CSD contain HMGB1. **A** Western blots of cortical EV lysates isolated with a commercial kit validated that the vesicles contained HMGB1 in addition to the EV marker protein TSG101 and neuronal marker CD171. Neuronal EVs subsequently separated with CD171-conjugated beads contained higher amounts of HMGB1. Western blots of neuronal EVs confirmed that they do not contain astrocyte- (ALDH1L1, S100B) and microglia-specific (Iba-1) markers, unlike the brain lysate used as a positive control. **B** The brain harvesting procedure itself caused considerable vesicular HMGB1 release, as seen in Western blots of the sham groups. However, HMGB1 in EVs was significantly higher in the CSD group than in the sham surgery group (Mann‒Whitney U test, *p* = 0.038). In the graph, each dot represents an individual mouse cortex subjected to optogenetically or pinprick-induced CSD (purple and pink dots, respectively). Whole membrane images of the Western blots are given in Additional file 2: Fig. S2.

Although these histological observations suggest that HMGB1 is released from neurons by way of EVs, they do not provide hints about the magnitude of this mechanism. To gain insight, we isolated EVs from the extracellular milieu of mouse brains one hour after CSD induction from both wild-type Swiss albino (by pinprick) and ChR2-eYFP transgenic mice (by optogenetic stimulation) and compared them to their sham-operated littermates. Based on the histological data, we anticipated that there would still be HMGB1-containing vesicles in the extracellular milieu one hour after CSD. We isolated all EVs irrespective of their cellular origin (i.e., neuronal and nonneuronal) with a commercially available kit in the initial set of experiments. For this, we first optimized the isolation conditions in preliminary experiments with the help of flow cytometry and NTA. Procedure-induced EV release (due to hypoxia/ischemia, etc., during brain harvesting) was minimized with optimizations (details can be found in the Methods section). NTA by the tunable resistive pulse sensing method validated that these isolated particles were EVs (1.39e + 10 particles/ml), and approximately 70% of them had a size compatible with exosomes (40–160 nm) [58] (Fig. 5A). This was also confirmed by SEM (Fig. 5B). Isolated EVs were captured by beads coated with anti-CD63, and anti-CD81 antibodies, the cardinal EV surface markers, and then evaluated by flow cytometry using fluorescent-tagged antibodies directed to CD63, and CD81 (Fig. 5C). We also showed by Western blotting that EV lysates contained HMGB1 in addition to the EV marker proteins TSG101 and CD171 (Fig. 6A). Next, we selectively separated EVs released from neurons by capturing them with beads coated with antibodies against the neuron-specific EV surface protein CD171. Western blots of neuronal EVs revealed that they contained large amounts of HMGB1 along with TSG101 and neuron (CD171) but not astrocyte- (ALDH1L1, S100B) and microglia-specific (Iba1) markers (Fig. 6A). We obtained similar results when CSD was triggered with relatively less invasive optogenetic stimulation in ChR2-expressing mice, confirming one more time that vesicular HMGB1 release was not caused by pinprick-induced trauma but by CSD (Fig. 6B). However, EVs from the sham-operated control group also contained HMGB1 (mean intensity 1 and 1.86 AU, *n* = 5 and 4 for the sham and CSD groups, respectively; *p* = 0.038, Mann‒Whitney U test), suggesting that by the time the hypoxia/ischemia-induced injurious brain events accompanying brain extraction, cell dissociation and EV isolation procedures had been completed, neurons and nonneuronal brain cells had already released significant amounts of HMGB1 despite measures taken to minimize this (e.g., cooling), as previously reported by several laboratories under hypoxic and ischemic conditions in the brain and other tissues [59–61].

## Discussion

We found that HMGB1 is released within sEVs from neurons after CSD-induced cellular stress. This is not surprising given that the HMGB1 protein does not have a leader peptide sequence to cross the plasma membrane by conventional protein secretion mechanisms [62, 63]. Although HMGBs were not detected in EVs obtained from several cell lines in vitro [64], this was not the case for challenged neurons in vivo, similar to HMGB1-containing EVs obtained from stimulated macrophages and hepatocytes [47, 65–67]. EM data conform with published studies [58, 68, 69] and suggest that HMGB1 is incorporated into sEVs’ complex cargo through conventional endosomal mechanisms and released into the extracellular medium within sEVs possibly following the fusion of multivesicular bodies transporting HMGB1-loaded sEVs to the plasma membrane. The EV size observed with EM was within the exosome range [58], which was confirmed by evaluating 10 billion EVs with NTA, suggesting that HMGB1 is largely released with exosomes. Furthermore, we did not observe plasma membrane blebbing suggestive of microvesicle formation with EM [58]. Although these data show that HMGB1 is released within sEVs, additional mechanisms may emerge when cellular stress is further increased because a previous study detected HMGB1 in CSF collected for one hour after multiple (but not single) CSDs, suggesting that, at least after recurrent neuronal stress, part of the released HMGB1 was transported to the CSF [7]. A small drop in cortical HMGB1 levels (i.e., loss of HMGB1 from brain tissue) as detected by Western blotting 3 h after multiple (but not single) CSDs supports this possibility [8] and suggests that recurrent CSD-induced stronger cellular stress may increase free HMGB1 in the interstitium. Indeed, a rise in CSF HMGB1 levels has also been reported after metabolic stress induced by pharmacological inhibition of brain glycogen utilization [70]. Secretory lysosomes activated for recycling internalized/damaged macromolecules by severe cellular stress [71] can liberate free HMGB1 to the extracellular medium upon fusion with the plasma membrane [1, 45]. Supporting this view, lysosomes have been shown to be activated with multiple CSDs [72].

We have also found that EVs harboring HMGB1 are taken up by astrocyte processes, suggesting that HMGB1 signaling is compartmentalized after a single CSD-induced cellular perturbation. Astrocytic EV uptake was visible in EM sections, whereas immunohistochemistry illustrated significantly different colocalization scores of the GFAP and HMGB1 signals between brain sections prepared from hGFAP-GFP transgenic mice subjected to CSD or sham procedures, suggesting that this mechanism is not uncommon. These observations are consistent with a study showing miRNA124-3P transport from neurons to astrocytes by way of EVs [73]. HMGB1’s role in initiating inflammation under various conditions is well characterized, and it is generally assumed that it binds to toll-like and RAGE receptors on the surface of nearby cells after being released into the interstitium [2]. This view originates mainly from studies in which the synthetic HMGB1 protein was directly applied to the culture medium of various cells [1, 74, 75]. However, if HMGB1 is released within EVs in vivo as previously predicted [2, 76] and shown in the present study, binding to intracellular counterparts of these receptors is more likely after uptake. The width of the interstitium around cell bodies ranges from 38 to 64 nm in the rat brain, smaller than most sEVs, creating an optimal environment for sEVs released to directly contact the large astrocyte membrane surface, ensheathing neuron soma [77] at the moment they are released, which may also be facilitated with more specific uptake mechanisms [71].

Astrocyte processes near the neuronal soma contain the endoplasmic reticulum, which connects to the endolysosomal system in peripheral branches that extend to the root branches and finally the soma. Although not investigated in this study, it is possible that the binding of HMGB1 to TLRs within the endolysosomal system [78] can initiate inflammatory signaling through various mechanisms, among which translocation of the proinflammatory transcription factor NF-ĸB p65 to the nucleus is well characterized [79]. Consistent with this formulation, NF-ĸB p65 is translocated to astrocyte nuclei after CSD but, interestingly, not in microglia. The essential role of HMGB1 in initiating nuclear NF-ĸB p65 translocation in astrocytes was shown by silencing HMGB1 expression as well as BoxA (a fragment of HMGB1 with antagonistic activity) and an antibody to HMGB1 in previous studies from our laboratory [7]. In the present study, we specifically investigated microglial activation and found that microglia displayed no inflammatory morphological features or change in Iba1 immunoreactivity or NF-ĸB p65 translocation to the nucleus although these observations do not exclude the pro/anti-inflammatory transcriptional changes induced by mechanisms other than HMGB1-NF-ĸB pathway. This observed discrepancy conforms to previous CSD studies, which consistently reported that microglia are only activated 24 h after multiple CSDs, but not after a single CSD [8, 42–44, 54, 80]. The extensive and relatively stable coverage of the neuron soma by astrocyte processes compared to highly mobile contacts by microglial processes may be one of the reasons for this cellular selectivity. This kind of compartmentalized (initially astrocyte-mediated) response to HMGB1 may be a prerequisite to controlled transcellular inflammatory signaling in response to a single CSD or similar transient CNS perturbations without escalating to the full-blown inflammation seen in overt inflammatory pathologies, in which free HMGB1 in the interstitium forms highly inflammatory heterocomplexes with other extracellular DAMPs before being endocytosed [1, 27, 81]. Likewise, increasing free HMGB1 levels in the interstitium during repeated CSDs over the course of an hour may activate the TLRs and RAGE receptors on the microglial plasma membrane. Although microglia have been reported to be necessary to achieve a significant level of NF-ĸB activation in astrocytes after exposure to HMGB1 in cell culture studies [82], this appears to be not the case after CSD in vivo, possibly due mainly to vesicular release of HMGB1. Parallel to our findings, HMGB1 release from neurons was reported to activate TLR4 in astrocytes but not microglia in mouse models of seizures, as well as specimens from patients with temporal lobe epilepsy with hippocampal sclerosis [83].

To our knowledge, this is the first demonstration of vesicular HMGB1 release from cortical neurons and their uptake by perisomatic astrocyte processes in vivo after noninjurious cellular stress. The tightly compartmentalized signaling between neurons and astrocyte processes separated from each other by an interstitium smaller than most EVs may serve to deliver the inflammatory message to the meninges from stressed neurons without putting the brain tissue at risk of overt inflammation. We propose that this signaling pathway may be an important alarm system for detecting and reporting transient brain perturbations such as migraine aura and epileptic discharges, provided that the inflammatory signal is strong enough to pass the pain threshold and not suppressed by central anti-nociceptive mechanisms [84–86]. Furthermore, this formulation also suggests that if such perturbations are prolonged or frequently repeated, they bear the potential to induce inflammation (e.g., by promoting the inflammatory microglial phenotype) and inflict tissue injury. Finally, these data raise the exciting possibility that HMGB1 antagonists such as glycyrrhizin and several anti-inflammatory and neuroprotective endogenous mediators such as endocannabinoids might prevent both headaches and unwanted inflammatory reactions to functional perturbations in nondegenerating CNS disorders [87–89].

### Supplementary Information

**Additional file 1: Figure S1. **Gating strategy applied to flow cytometry. Forward and side scatter gating is used in flow cytometry analysis to identify the single beads based on the relative size and complexity (clumping) of the beads while removing debris and other events that are not of interest. The mean fluorescence intensity was used as a quantitative measure.

**Additional file 2: Figure S2. **Original Western blot membrane images. A. Whole membrane images of Fig. 6A, upper left panel. B. Whole membrane images of Fig. 6A, upper right panel. Bands of interest are marked with red arrows in A&B. C. Whole membrane images of Fig. 6A, lower panel. Detailed information can be found in the legend of original figure.

## Data Availability

All data associated with this study are presented in the paper or the Supplementary Materials.

## Abbreviations

HMGB1: High mobility group box 1
CSD: Cortical spreading depolarization
sEV: Small extracellular vesicles
MA: Migraine with aura
PAMP: Pathogen-associated molecular pattern
DAMP: Damage-associated molecular pattern
DC: Direct current
NTA: Nanoparticle tracking analysis
MIP: Maximum intensity projection
TOS: Threshold overlap score
TLR: Toll-like receptors
