# Supplementary figures and images for: Vesicular HMGB1 release from neurons stressed with spreading depolarization enables confined inflammatory signaling to astrocytes
Source: J Neuroinflammation. 2023 Dec 11;20:295. doi: 10.1186/s12974-023-02977-6 (PMC10712196; doi:10.1186/s12974-023-02977-6)

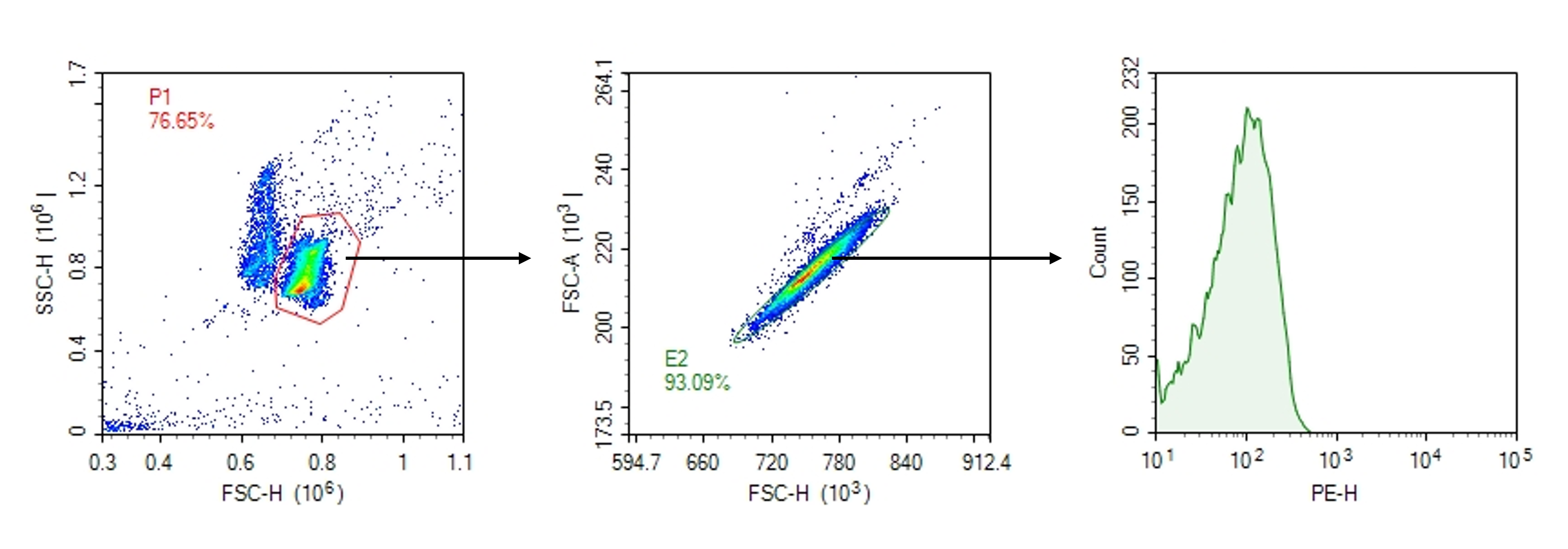

Supplement: Supplementary file 1 — Additional file 1: Figure S1. Gating strategy applied to flow cytometry. Forward and side scatter gating is used in flow cytometry analysis to identify the single beads based on the relative size and complexity (clumping) of the beads while removing debris and other events that are not of interest. The mean fluorescence intensity was used as a quantitative measure. [file 12974_2023_2977_MOESM1_ESM.tif]

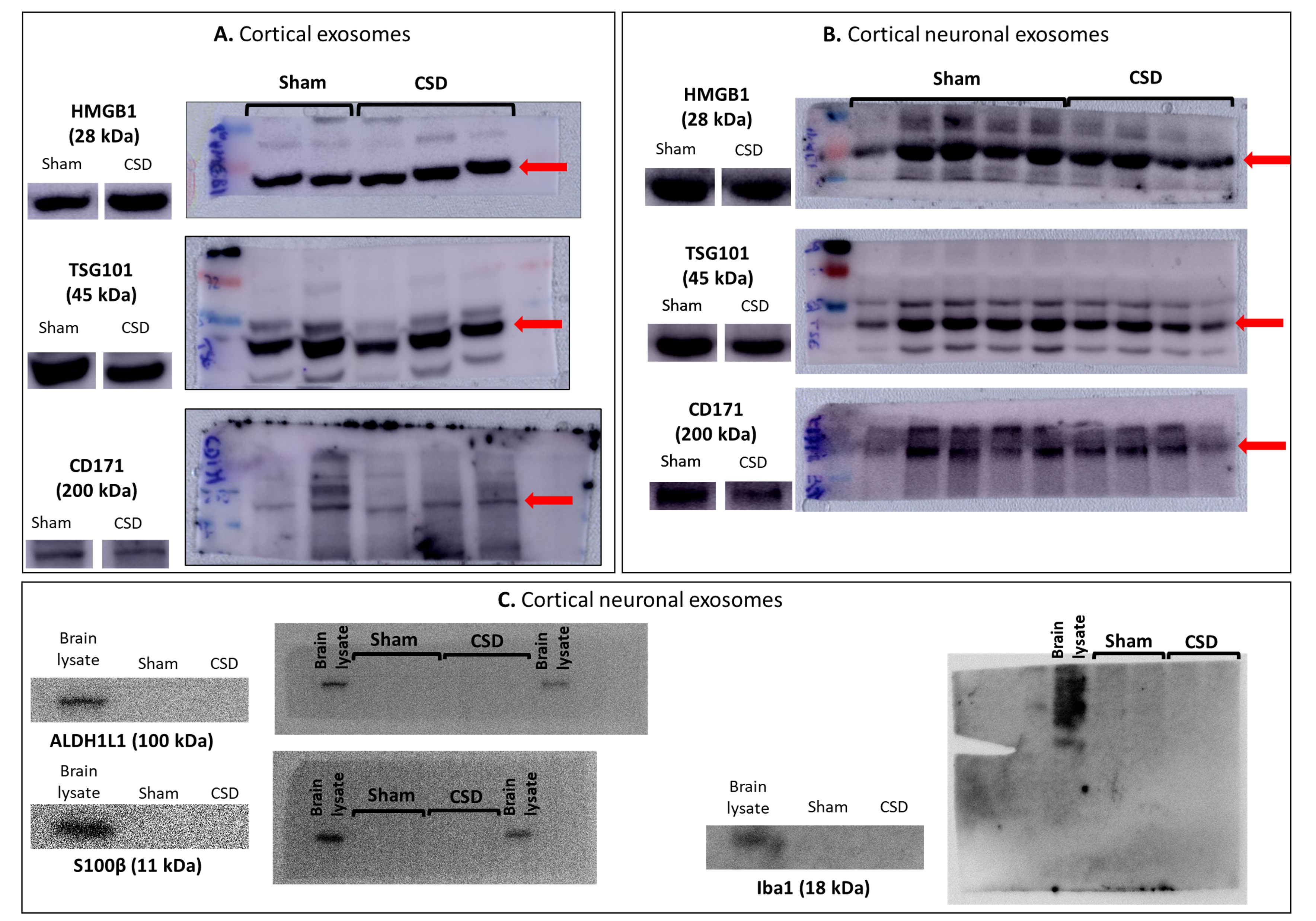

Supplement: Supplementary file 2 — Additional file 2: Figure S2. Original Western blot membrane images. A. Whole membrane images of Fig. 6A, upper left panel. B. Whole membrane images of Fig. 6A, upper right panel. Bands of interest are marked with red arrows in A&B. C. Whole membrane images of Fig. 6A, lower panel. Detailed information can be found in the legend of original figure. [file 12974_2023_2977_MOESM2_ESM.tif]
